# Analysis of the California list of pesticides, mycotoxins, and cannabinoids in chocolate using liquid chromatography and low‐pressure gas chromatography‐based platforms

**DOI:** 10.1002/jssc.202001265

**Published:** 2021-05-24

**Authors:** Nathaly Reyes‐Garcés, Colton Myers

**Affiliations:** ^1^ Department of Research & Development Restek Corporation Bellefonte Pennsylvania USA

**Keywords:** California pesticides and mycotoxins, cannabinoids, cannabis, chocolate, low‐pressure gas chromatography

## Abstract

Cannabis legalization has led to the development of a variety of cannabis‐infused products with edibles being one of the most popular. The state of California has implemented comprehensive cannabis testing regulations requiring the analysis of cannabinoids (potency) and contaminants, such as pesticides and mycotoxins, in any type of cannabis good. In this work, we propose an analytical workflow for the quantification of the California list of pesticides and mycotoxins, as well as six cannabinoids, in chocolate, using 3 mL of solvent for the extraction. For the analysis of pesticides and mycotoxins, clean‐up steps employing a C18 solid‐phase extraction cartridge and dispersive solid‐phase extraction sorbents were implemented. Gas chromatography amenable pesticides were analyzed using low‐pressure gas chromatography coupled to tandem mass spectrometry which allowed for a total method run of 12 min. Both liquid chromatography and gas chromatography instrumental methods had the same analysis time, ensuring satisfactory sample throughput. For the determination of cannabinoids, a dilution of the original organic extract collected for pesticides and mycotoxins analysis (and prior to any clean‐up step) was used. Excellent results in terms of analytical figures of merit were obtained for all target analytes.

**Article Related Abbreviations**
AOAC: Association of Official Analytical Chemists
CBD: cannabidiol
CBDA: cannabidiolic acid
CBG: cannabigerol
CBN: cannabinol
d9‐THCA‐A: delta 9‐tetrahydrocannabinolic acid A
delta 9‐THC: delta 9‐tetrahydrocannabinol
dSPE: dispersive solid‐phase extraction
EI: electron ionization
ISTDs: internal standards
LPGC: low pressure‐gas chromatography
PSA: primary secondary amine
QuEChERSER: quick, easy, cheap, effective, rugged, safe, efficient, and robust
SMPRs: standard method performance requirements

## 1 INTRODUCTION

Legalization of cannabis for medical and adult use has led to the development of a plethora of products to satisfy cannabis consumers. Currently, 36 states and the District of Columbia allow for the usage of medicinal cannabis while 11 states allow the use of low delta 9‐tetrahydrocannabinol (delta 9‐THC) and high cannabidiol (CBD) products for medical reasons in limited situations or as a legal defense, and 15 states and the District of Columbia legalized the use of cannabis for adult use [1]. Hence, the availability and diversity of cannabis goods may vary depending on the state. Cannabis testing regulations also differ depending on each state law. The main analytical tests required from third party labs can include the analysis of phytoconstituents (e.g. cannabinoids and terpenes), contaminants such as microbial impurities, mycotoxins, pesticides, heavy metals, and residual solvents. While the analysis of contaminants is required only in the plant material in the majority of states, in California it is mandatory to test for these contaminants in all cannabis goods [2]. Undoubtedly, obtaining reliable analytical data is a challenging undertaking due to the broad variety of analytes and cannabis matrices. Hence, that robust methodologies capable of providing trustworthy results in a simple, fast, and cost‐effective fashion are highly desired.

Relevant studies seeking to address the need of reliable testing methods in cannabis and its derived products have been published over the last years [3, 4, 5, 6, 7, 8, 9]. With regards to pesticides testing, the great majority of the works have been focused on plant material [4, 5, 6, 10], followed by some studies on cannabis oils [11, 12, 13]. It is worth mentioning that a stake holder panel on strategic food analytical methods recently published in the Journal of the Association of Official Analytical Chemists (AOAC), a set of standard method performance requirements (SMPRs) describing the minimum recommended performance criteria for any method to be applied in the analysis of pesticides in dried cannabis [14]. Such guidelines provide information on recommended LOQs, recoveries, and precision. Common sample preparation strategies for the isolation of pesticides from diverse cannabis products involve solvent extraction followed by extract dilution; QuEChERS; and SPE as an extract clean‐up step [6, 11, 15, 16, 17, 18]. An updated version of QuEChERS known as QuEChERSER mega method, which stands for quick, easy, cheap, effective, rugged, safe, efficient, and robust, was recently applied for the analysis of a broad range of pesticides in hemp matrices [10]. Other approaches involving SPME and direct coupling of SPME‐based devices with tandem mass spectrometry (MS/MS) have also been evaluated, and satisfactory results have been found for a limited number pesticides [12, 19]. As for instrumental analysis, the use of LC coupled to MS/MS is currently the technique of choice due to selectivity, sensitivity, and suitability to detect the great majority of regulated pesticides in USA and Canada [6]. To account for regulated pesticides that are not easily ionized via ESI, the use of either LC‐MS/MS with alternative ionization mechanisms, such as APCI, or more traditional instrument approaches, such as GC‐MS/MS, which use electron ionization (EI), have been adopted as a common practice [6, 15]. Considering the long run times typically associated with the analysis of GC‐amenable pesticides in complex matrices, an alternative approach known as low‐pressure GC (LPGC) has been reported in the analysis of diverse food commodities [20, 21]. LPGC is a concept that was introduced in the 1960s by Giddings where the GC column outlet is placed under vacuum conditions to speed up the time of analysis [22]. A decrease in the carrier gas viscosity generated by dropping the column pressure shifts the optimum average linear flow velocity in the van Deemter equation toward higher flow rates. Under these conditions, faster separations can be attained while maintaining the same degree of resolution [20, 23]. It should be noted that LPGC‐MS/MS was recently used for the determination of multiple GC amenable pesticides regulated by USA and Canada in hemp products extracts obtained via QuEChERSER and after automated robotic minicolumn SPE cleanup [10]. The results of this study demonstrated the potential of LPGC‐MS/MS for faster analysis of GC‐amenable contaminants in cannabis products.

Several reports on the analysis of mycotoxins in cannabis plants and derived products have been published as well [24, 25, 26, 27]. Although determination of mycotoxins by employing immunoaffinity columns in combination with LC coupled to fluorescence detection is a valid approach, quantitation of both pesticides and mycotoxins from the same sample extract and under the same LC‐MS/MS method conditions is the most popular strategy [3, 15, 24].

In terms of phytoconstituents, multiple studies describing diverse analytical methods for the determination of cannabinoids and terpenes in cannabis matrices have been recently reported [28, 29, 30, 31, 32, 33, 34, 35, 36, 37]. Furthermore, SMPRs for the quantitation of cannabinoids in cannabis concentrates have been published [38], and several literature reviews outlining the most up‐to‐date information in terpene and potency analysis are also available [7, 8, 39, 40]. Generally, potency testing is conducted by performing a solvent extraction, followed by a dilution/centrifugation step, and LC‐ultraviolet detection (UV) analysis. Considering the broad variety of cannabis products available, different sample treatment steps may be required in order to homogenize and successfully extract cannabinoids of interest from various matrices.

The goal of this study was to introduce an effective workflow for the analysis of pesticides, mycotoxins, and cannabinoids in chocolate, a popular cannabis‐infused edible that can be considered a complex matrix due to its high content of fat and sugars. Currently, there are not reported methods for the analysis of these three compound classes in cannabis‐infused chocolate, and only few studies have investigated the determination of cannabinoids in this cannabis edible [31, 32, 41]. For the analysis of pesticides, two instrumental platforms would be used: LC‐MS/MS and LPGC‐MS/MS. For the analysis of cannabinoids, the same organic extract collected for analysis of pesticides and mycotoxins would be run under LC‐UV conditions.

## 2 MATERIALS AND METHODS

### 2.1 Standards and chemicals

The following certified reference standards were obtained from Restek Corporation (Bellefonte, PA, USA): pesticides mixes (6) corresponding to the California list at 100 μg/mL in acetonitrile (additional information about the mixes composition is provided in the supporting information); cannabinoid standards at a concentration of 1000 μg/mL in either methanol or acetonitrile (cannabidiolic acid [CBDA], cannabigerol [CBG], delta 9‐tetrahydrocannabinolic acid A [d9‐THCA‐A], and a mix of cannabinol (CBN), delta 9‐THC, and CBD); 10 ppm aflatoxin mix in acetonitrile (aflatoxins B1, B2, G1, G2); ochratoxin A standard at 10 ppm in acetonitrile; and 100 μg/mL solutions of seven deuterated analogues in either acetonitrile or acetone (dimethoate‐d6, dichlorvos‐d6, carbaryl‐d7, diazinon‐d10, atrazine‐d5, diuron‐d6, and linuron‐d6). A certified standard of daminozide‐d6 was purchased from LGC (Manchester, NH). LC‐MS grade water, acetonitrile, methanol, and MS compatible ammonium formate, acetic acid, and formic acid were purchased from Fisher Scientific.

For sample preparation, Resprep 100 mg C18 SPE cartridges, tubes with preweighed dispersive SPE (dSPE) sorbents (150 mg of magnesium sulfate and 25 mg of primary secondary amine [PSA]), 4 mL vials, SPE vacuum manifold, quick‐replace disposable liners, and autosampler vials were all obtained from Restek Corporation.

Blank chocolate matrix and CBD‐infused chocolate (1 mg/g of CBD) were purchased from a local store.

### 2.2 Sample preparation conditions

**FIGURE 1:**
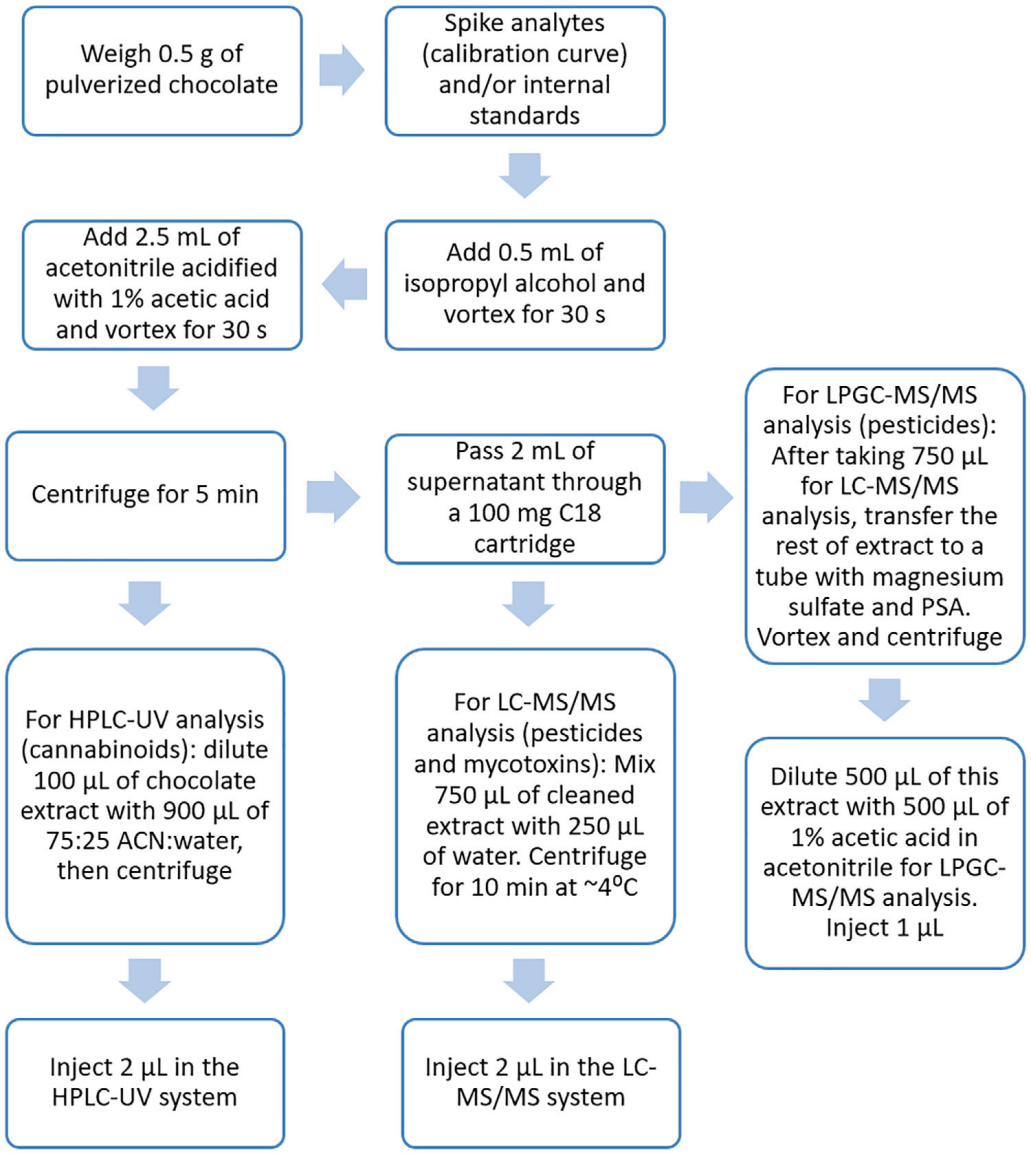
Sample preparation workflow for the analysis of pesticides, mycotoxins, and cannabinoids in chocolate samples

Chocolate samples were pulverized using either a freezer mill from SPEX (Metuchen, NJ) (for pesticides and mycotoxins method development) or a food processor with dry ice (for potency testing and final method validation, as the freezer mill was unavailable for this part of the work). A sum of 0.5 g of sample was weighed in a 4 mL glass vial. Analytes and/or internal standards (ISTDs) were spiked in the dry matrix. After waiting for 10 min to allow analytes time to bind to matrix components, 0.5 mL of isopropyl alcohol was added to the vial. The samples were vortexed for 10 s, or until obtaining a homogenous mixture. Afterwards, 2.5 mL of acetonitrile acidified with acetic acid at 1% v/v was added to the vial. Once again, the mixture was vortexed for 30 s, and then centrifuged for 5 min at 4300 × *g* at room temperature. A sum of 2 mL of the supernatant was passed through Resprep 100 mg C18 cartridge. For LC‐MS/MS analysis, 750 μL of clean extract was mixed with 250 μL of water, and the mix was centrifuged for 10 min at 4°C to precipitate undissolved fat. For LPGC‐MS/MS analysis, the remaining supernantant was subjected to an extra clean‐up step using dSPE sorbents (magnesium sulfate and PSA). After vortexing and centrifuging, 500 μL of extract was diluted with 500 μL of acetonitrile acidified at 1% v/v with acetic acid. Figure 1 summarizes the sample preparation steps followed to analyze chocolate samples. It is worth emphasizing that blank chocolate samples were analyzed to ensure that they were free of target contaminants.

Cannabinoids were analyzed by taking an aliquot of the chocolate extracts collected for pesticides and mycotoxins analysis immediately after centrifugation and prior to any clean‐up step with SPE or dSPE sorbents. For analysis of cannabinoids in chocolate using LC‐MS/MS, 20 μL of extract was diluted to 1000 μL with acetonitrile, and this solution was further diluted by mixing a 100 μL aliquot with 900 μL of acetonitrile. For HPLC‐UV testing, 100 μL of chocolate extract was mixed with 900 μL of 75:25 acetonitrile:water, and the final mix was centrifuged for 10 min at 4°C.

### 2.3 Instrumental analysis

#### 2.3.1 LC/LPGC‐MS/MS conditions for the analysis of pesticides, mycotoxins, and cannabinoids

For LC‐MS/MS analysis, a Shimadzu 8060 (Kyoto, Japan) triple‐quadrupole system coupled to a Nexera UHPLC pump and a SIL‐30AC autosampler were used. Analyte separation was achieved through the use of a Raptor ARC‐18 column (2.1 × 100 mm, 2.7 μm particle size) connected to a Raptor ARC‐18 EXP guard column cartridge 5 mm, 2.1 mm ID, 2.7 μm from Restek Corporation. The mobile phases used were water with 2 mM ammonium formate and 0.1% formic acid (A) and methanol with 2 mM ammonium formate and 0.1% formic acid (B). Mobile phase gradient conditions were as follows: linear increase from 5% B to 65% B in 1.5 min, linear increase of B to 95% in 7 min, and then increase of B to 100% in 1 min. Hold at 100% B for 1 min, and finally the column was re‐equilibrated for 2 min at 5% B. The total run time was 12 min, the flow rate was 0.5 mL/min and the column temperature was maintained at 40℃ . The injection volume was 2 μL and the autosampler temperature was kept at 10℃. MS conditions were set as follows: spray voltage = 4 kV, nebulizing gas flow = 3 L/min, heating gas flow = 10 L/min, drying gas flow = 10 L/min, interface, desolvation line and heat block temperatures were all set at 100°C. Samples were run in alternating positive and negative multiple reaction monitoring mode. Optimum MS/MS conditions (Q1 and Q3 Pre Bias, and collision energies) were determined for each compound by using direct infusion of standards (Supporting information Table S1). Due to the multiple transitions monitored in this particular method, MS data were collected by defining acquisition windows based on chromatographic retention time. LabSolutions (version 5.89) and LabSolutions Insight (version 3.2) (Shimadzu) were utilized for data acquisition and processing. Supporting information Figure S1 shows a representative chromatogram of the LC‐MS/MS amenable pesticides and mycotoxins.

For the experiments where cannabinoids were analyzed using LC‐MS/MS, a Raptor ARC‐18 column (2.1 × 100 mm, 2.7 μm particle size) connected to a Raptor ARC‐18 EXP guard column cartridge 5 mm, 2.1 mm ID, 2.7 μm from Restek Corporation. The mobile phases used were water with 5 mM ammonium formate and 0.1% formic acid (A) and acetonitrile with 0.1% formic acid (B). Samples were run at isocratic conditions, holding B at 75% with a flow rate of 0.4 mL/min for 10 min in each injection [42]. The column temperature was kept at 30°C and the injection volume was 1 μL. MS conditions were set as follows: spray voltage = 4 kV, nebulizing gas flow = 3 L/min, heating gas flow = 10 L/min, drying gas flow = 10 L/min, interface, DL and heat block temperatures were all set at 100°C. Information about MS/MS transitions can be found in the Supporting information Table S2.

Analysis of seven pesticides and two ISTDs was performed by using LPGC‐MS/MS on a Thermo Trace 1310 GC coupled to a TSQ 8000 triple quadrupole mass spectrometer with EI (Thermo Scientific, San Jose, USA). Analytes were separated using an Rxi‐5 ms, 15 m × 0.53 mm × 1.00 μm, connected to a Hydroguard column, 5 m × 0.18 mm (Restek Corporation). Supporting information Tables S3 and S4 show LPGC‐MS/MS method parameters and the MS/MS transitions monitored, respectively. A chromatogram displaying the compounds that were analyzed via GC‐MS/MS is shown in Supporting information Figure S2.

### 2.4 HPLC‐UV conditions for the analysis of cannabinoids

For the analysis of cannabinoids using HPLC‐UV, a Waters Acquity PDA system was used (Milford, MA, USA). The same LC gradient described in the previous section was applied. Sample injection volume was set at 2 μL and the UV wavelength was set at 228 nm. Data were acquired and processed using Empower 3 by Waters (Milford). Supporting information Figure S3 presents a chromatogram obtained in matrix displaying the target cannabinoids analyzed via HPLC‐UV.

### 2.5 Recoveries assessment

For pesticides and mycotoxins, recoveries were assessed by comparing analyte responses corresponding to extracts obtained from chocolate samples (0.5 g) prespiked at 100 ng/g (n = 3) versus responses from 3 mL of chocolate blank extracts postspiked at 16.6 ng/mL (n = 3) and further diluted in a ratio 75:25 extract:water for instrumental analysis. This experiment was carried out using two extraction solvents: 3 mL of acetonitrile acidified at 1% with acetic acid (v/v) and 0.5 mL of IPA and 2.5 mL of acetonitrile acidified at 1% with acetic acid (v/v).

Cannabinoid recoveries were initially tested by spiking chocolate with CBD, CBN, and delta 9‐THC at a concentration of 0.6 mg/g. For this purpose, 300 μL of a 1000 μg/mL standard containing the three cannabinoids were added to blank chocolate samples (0.5 g, n = 3). Extraction conditions were as described in the sample preparation conditions section, and extracts were run under LC‐MS/MS conditions. Recoveries were estimated by running a blank chocolate extract postspiked at the same concentration level. To investigate the effect of extraction solvent volume in cannabinoid recovery, 0.5 g samples of pulverized CBD‐infused chocolate (1 mg/g) were extracted with 3 mL (0.5 mL IPA/2.5 mL acidified acetonitrile), 6 mL (1 mL IPA/5 mL of acidified acetonitrile) and 12 mL (2 mL of IPA/10 mL of acidified acetonitrile) of solvent. Recoveries were calculated by running a CBD calibration curve prepared in solvent covering a concentration range from 10 to 500 ppb. Extracts corresponding to this experiment were run under LC‐MS/MS conditions. Estimated concentrations were compared against the product label.

### 2.6 Linearity, accuracy, precision, and LOQs

For pesticides quantification, calibration curves were obtained by spiking target analytes in blank matrix at eight different concentration levels (5, 10, 25, 50, 75, 200, 400, and 700 ng/g) (n = 2). In the case of mycotoxins, the spiking levels were 5, 10, 25, 40, 75, and 150 ng/g (n = 2). Calibration curves were then constructed by plotting analyte area/ISTD area ratios versus spiked concentration. At least six concentration levels were used to construct each curve and a weighing factor of 1/× was applied in all the cases. Accuracy and precision were assessed at three concentration levels (low: 10 ng/g; medium: 100 ng/g; high: 500 ng/g) in quadruplicates. For mycotoxins, the concentration levels chosen to test accuracy and precision were 10 ng/g (low), 50 ng/g (medium), and 100 ng/g (high). LOQs were determined as the lowest concentrations with a *S/N* ratio of at least 10, a difference of less than 25% between the nominal concentration and the calculated concentration, and a RSD value of less than 25%.

For the analysis of cannabinoids using HPLC‐UV, calibration points were prepared in 75:25 acetonitrile:water in a concentration range spanning from 2 to 200 ppm. Calibration curves were built by plotting analyte concentrations versus area counts. A weighing factor of 1/*x* was applied. Determination of method accuracy and precision was assessed by spiking chocolate samples at 0.2, 0.5, and 1 mg/g (n = 3), and by comparing the estimated concentration versus nominal concentration of analytes spiked.

### 2.7 Assessment of ESI ionization effects

Absolute matrix effects for the LC amenable pesticides were investigated by following the procedure proposed by Matuszewski et al. [43]. Briefly, blank chocolate extracts spiked at a concentration of 16.6 ng/mL were compared against neat solvent spiked at the same concentration level. Matrix effects were estimated by using the following equation: (analyte response in blank extract/analyte response in neat solvent) × 100.

### 2.8 Assessment of extracts stability

Stability of extracts used for pesticide and mycotoxin analysis was investigated by comparing the responses of freshly prepared extracts obtained from chocolate samples spiked at 100 ng/g versus the same samples after 24 and 48 h in the autosampler trays. For the LC amenable analytes, samples were stored at 10°C. Extracts for GC analysis were kept at room temperature.

## 3 RESULTS AND DISCUSSION

### 3.1 Analysis of pesticides and mycotoxins

#### 3.1.1 Evaluation of extraction recoveries

**FIGURE 2:**
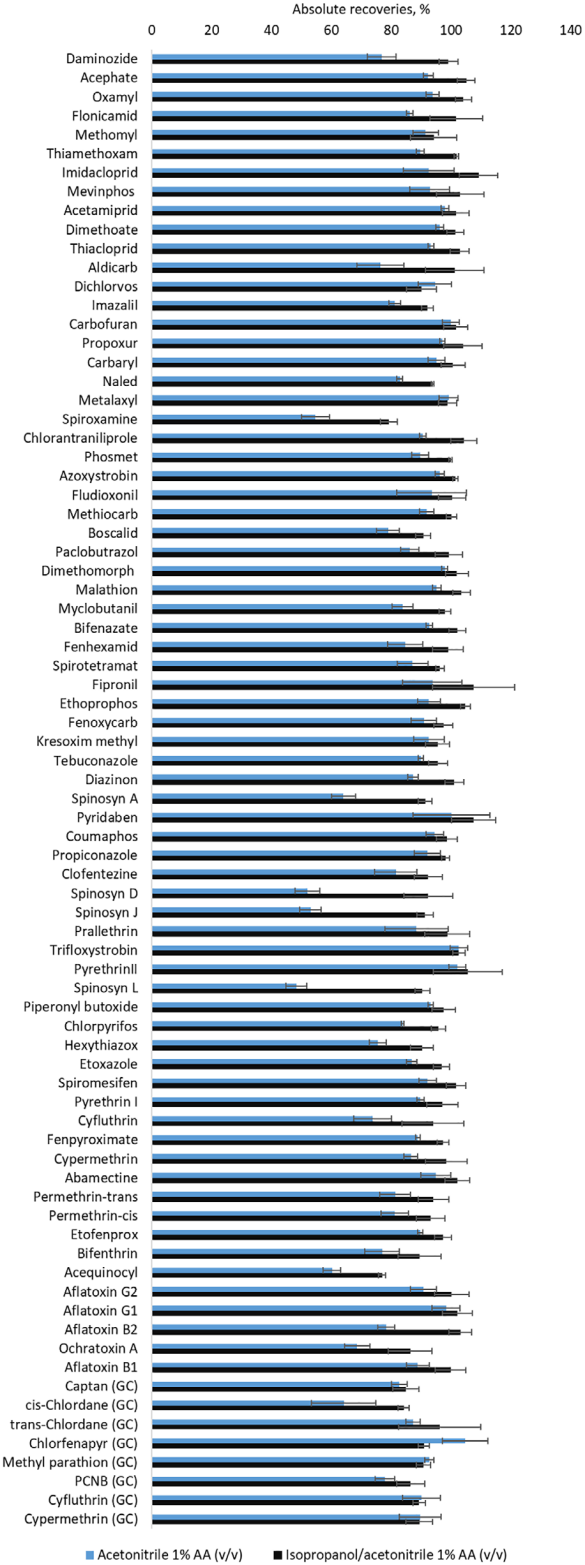
Absolute recoveries corresponding to contaminants spiked in dry chocolate samples at 100 ng/g (n = 3). Analyte responses obtained from prespiked extracts were compared against peak areas corresponding to postspiked chocolate extracts (n = 3)

First, a comparison between experimental conditions previously optimized for the analysis of pesticides and mycotoxins in brownies [44], and a modified procedure where IPA (0.5 mL) and acetonitrile acidified with 1% v/v acetic acid (2.5 mL) were used as extraction solvents (refer to the Materials and Methods section) was conducted. Briefly, extraction of the California list of pesticides and mycotoxins from brownies involved weighing 0.5 g of pulverized sample, adding in two steps (1.5 mL each time) 3 mL of acetonitrile acidified with 1% v/v acetic acid, vortexing, centrifuging, and then cleaning up the resulting supernatant by passing it through a 100 mg C18 cartridge. Chocolate extracts obtained with the two tested extraction solvents were also cleaned using a 100 mg C18 cartridge to remove major hydrophobic interferences coextracted from the matrix. For the analysis of LC amenable analytes, a centrifugation step at low temperature after mixing 750 μL of extract with 250 μL of water was performed to assist in the precipitation of fats that were not retained in the C18 cartridge. It is worth to emphasize that a high level of cloudiness was observed when water was added to chocolate extracts that were not previously cleaned with C18 cartridges. This confirms that the use of C18 assists in the removal of significant fat content coextracted from chocolate. In the case of GC amenable compounds, a clean‐up step using magnesium sulfate to remove water residues and PSA to remove sugars from extracts was added. As shown in Figure 2, the use of only acetonitrile led to recoveries under 70% for spiroxamine, spinosad (spinosyn A and D), spinetoram (spinosyn J and L), and acequinocyl. Based on their log P information [45] (refer to Table 1), these analytes are hydrophobic pesticides that can display high affinity for the fat content present in chocolate; hence, the use of a solvent, such as isopropanol, is required to effectively extract them from such type of edible. In addition, spiroxamine and spinosyns contain in their structure moieties are capable of interacting via hydrogen bonding with other highly abundant matrix components such as carbohydrates. Overall, our results demonstrated that the use of 3 mL of solvent to extract from 0.5 g of chocolate was sufficient to obtain recoveries above 70% for all the LC amenable contaminants and above 80% for all the pesticides that were analyzed under GC‐MS/MS conditions.

**TABLE 1:** Validation results for the optimized final method for the analysis of pesticides and mycotoxins in chocolate

				Low conc. (10 ng/g)		Medium conc. (100 ng/g)		High conc. (500 ng/g)	
Contaminants (log*P*)^a^	Action level, ng/g	LOQ, ng/g	*R* ^2^	Accuracy (%)	Precision, RSD (%)	Accuracy (%)	Precision, RSD (%)	Accuracy (%)	Precision, RSD (%)
LC‐Pesticides									
Daminozide (−1.14)	100	25	0.9937	–	–	90	2	97	4
Acephate (−0.85)	5000	25	0.9951	–	–	95	16	94	4
Oxamyl (−0.47)	200	5	0.9970	90	9	101	10	95	2
Flonicamid (0.84)	2000	25	0.9962	–	–	90	4	96	2
Methomyl (0.60)	100	5	0.9985	99	9	95	7	96	3
Thiamethoxam (−1.16)	4500	5	0.9983	106	6	100	9	96	4
Imidacloprid (−0.43)	3000	25	0.9955	–	–	103	10	97	3
Mevinphos (0.28)	100	50	0.9971	–	–	99	10	97	2
Acetamiprid (0.62)	5000	5	0.9975	99	6	102	11	95	2
Dimethoate (1.32)	100	5	0.9975	100	7	94	2	97	1
Thiacloprid (0.55)	100	5	0.9968	101	6	102	8	97	5
Aldicarb (1.13)	100	25	0.9921	–	–	101	9	101	5
Dichlorvos (0.71)	100	25	0.9968	–	–	94	7	99	1
Imazalil (3.58)	100	5	0.9971	92	20	98	4	97	3
Carbofuran (1.76)	100	5	0.9972	92	10	100	6	98	2
Propoxur (1.60)	100	5	0.9982	97	3	99	7	99	3
Carbaryl (3.35)	500	5	0.9987	100	9	93	4	95	6
Naled (1.86)	500	5	0.9962	93	11	101	2	100	3
Metalaxyl (2.15)	15 000	5	0.9977	100	14	95	5	97	2
Spiroxamine (4.88)	100	5	0.9990	95	10	96	11	97	5
Chlorantraniliprole (5.55)	40 000	25	0.9967	–	–	96	1	99	4
Phosmet (2.84)	200	5	0.9962	93	12	97	5	96	3
Azoxystrobin (5.13)	40 000	5	0.9972	105	4	99	3	98	2
Fludioxonil (3.67)	30 000	25	0.9927	–	–	98	8	96	4
Methiocarb (2.88)	100	25	0.9976	–	–	93	2	98	2
Dimethomorph (I and II) (3.71)	20 000	25	0.9956	–	–	94	3	101	3
Boscalid (4.31)	10 000	5	0.9960	99	8	96	3	100	2
Paclobutrazol (2.99)	100	25	0.9978	–	–	96	6	99	2
Malathion (2.92)	5000	10	0.9953	102	12	93	5	99	3
Myclobutanil (2.82)	9000	5	0.9967	99	16	94	4	98	3
Bifenazate (3.12)	5000	5	0.9951	102	9	88	5	98	3
Fenhexamid (4.02)	10 000	25	0.9955	–	–	95	3	99	4
Spirotetramat (4.59)	13 000	10	0.9961	97	9	96	6	97	2
Ethoprophos (3.59)	100	5	0.9969	98	10	97	7	98	4
Fipronil (4.76)	100	25	0.9900	–	–	88	7	95	2
Fenoxycarb (3.83)	100	5	0.9954	103	8	91	3	97	3
Kresoxim methyl (4.34)	1000	25	0.9963	–	–	99	3	100	3
Tebuconazole (3.58)	2000	5	0.9969	102	4	94	4	99	2
Diazinon (3.81)	200	5	0.9981	99	3	95	3	98	1
Spinosad‐ spinosyn A (71 %)^b^ (4.80)	^k^	18	0.9962	–	–	103	11	99	4
Coumaphos (3.86)	100	5	0.9970	108	8	91	3	99	2
Pyridaben (4.73)	3000	75	0.9963	–	–	92	6	100	3
Propiconazole (3.88)	20 000	25	0.9954	–	–	94	5	98	3
Clofentezine (3.27)	500	5	0.9972	93	10	95	5	102	3
Spinosad ‐ spinosyn D (29%)^c^ (5.39)	^k^	7.3	0.9973	103	10	102	8	97	3
Spinetoram ‐ spinosyn J (80%)^d^ (4.51)	^l^	4	0.9978	98	7	101	9	98	3
Trifloxystrobin (5.11)	30 000	5	0.9970	109	7	93	3	98	1
Prallethrin (4.38)	400	50	0.9960	–	–	93	5	99	5
Pyrethrin II (34%)^f^ (4.43)	^m^	17	0.9925	–	–	99	1	101	2
Spinetoram ‐ spinosyn L (20%)^e^ (NF)	^l^	5	0.9974	98	4	100	10	95	4
Piperonyl Butoxide (4.23)	8000	5	0.9985	97	8	100	4	96	4
Chlorpyrifos (4.77)	100	10	0.9965	114	9	89	5	98	3
Hexythiazox (3.41)	2000	25	0.9964	–	–	91	6	100	3
Etoxazole (5.85)	1500	5	0.9975	104	3	92	4	100	3
Spiromesifen (5.83)	12 000	25	0.9962	–	–	96	6	99	4
Pyrethrin I (54%)^g^ (5.49)	^m^	27	0.9937	–	–	94	2	98	3
Cyfluthrin (6.29)	1000	75	0.9971	–	–	94	12	96	9
Cypermethrin (6.27)	1000	50	0.9945	–	–	81	7	102	5
Fenpyroximate (6.44)	2000	5	0.9981	101	5	93	3	99	2
Permethrin‐trans (59%)^h^ (7.15)	^n^	15	0.9971	–	–	89	6	102	2
Permethrin‐cis (41%)^i^ (7.15)	^n^	10	0.9977	–	–	91	6	98	4
AbamectinB1a (6.51)	300	25	0.9931	–	–	97	9	101	4
Etofenprox (7.34)	100	5	0.9974	108	7	89	3	100	2
Bifenthrin (7.30)	500	25	0.9980	–	–	88	6	99	1
Acequinocyl (8.45)	4000	25	0.9944	–	–	85	7	101	4
									
LC‐Mycotoxins									
Aflatoxin G2^j^ (0.75)	^o^	10	0.9929	93	15	90	13	99	8
Aflatoxin G1^j^ (1.09)	^o^	5	0.9966	96	19	96	6	104	7
Aflatoxin B2^j^ (0.52)	^o^	5	0.9984	96	14	106	5	96	2
Aflatoxin B1^j^ (0.45)	^o^	5	0.9975	92	15	103	6	100	5
Ochratoxin A^j^ (4.31)	20	10	0.9881	113	13	103	13	104	8
									
LPGC‐Pesticides									
Pentachloronitrobenzene (GC) (4.16)	200	10	0.9951	104	8	95	9	104	7
Methyl parathion (GC) (2.78)	100	5	0.9976	110	5	97	4	103	3
Captan (GC) (1.85)	5000	25	0.9914	–	–	111	5	109	3
trans‐Chlordane (GC) (5.57)	^p^	50	0.9888	–	–	102	14	102	13
cis‐Chlordane (GC) (5.57)	^p^	50	0.9933	–	–	101	17	108	14
Chlorfenapyr (GC) (5.16)	100	10	0.9939	110	11	107	18	110	9
Cyfluthrin (GC) (6.29)	1000	5	0.9957	110	10	102	10	110	3
Cypermethrin (GC) (6.27)	1000	10	0.9967	115	8	100	13	109	3
Category I pesticides, LOQ ≤ 100 ng/g									

^a^ Log(*P*) values were taken from Chemspider [45].

NF, not found.

^b^ Spinosad‐spinosyn A: low: 7 ng/g; medium: 71 ng/g; high: 355 ng/g.

^c^ Spinosad‐spinosyn D: low: 3 ng/g; medium: 29 ng/g; high: 145 ng/g.

^d^ Spinetoram‐spinosyn J: low: 8 ng/g; medium: 80 ng/g; high: 400 ng/g.

^e^ Spinetoram‐spinosyn L: low: 2 ng/g; medium: 20 ng/g; high: 100 ng/g.

^f^ Pyrethrin I: low: 5 ng/g; medium: 54 ng/g; high: 270 ng/g.

^g^ Pyrethrin II: low: 3 ng/g; medium: 34 ng/g; high: 170 ng/g.

^h^ Permethrin‐cis: low: 4 ng/g; medium: 41 ng/g; high: 205 ng/g.

^i^ Permethrin‐trans: low: 6 ng/g; medium: 59 ng/g; high: 295 ng/g.

^j^ Mycotoxins: low: 10 ng/g; medium: 50 ng/g; high: 100 ng/g.

^k^ Total spinosad should not exceed 3000 ng/g.

^l^ Total spinoteram should not exceed 3000 ng/g.

^m^ Total pyrethrins should not exceed 1000 ng/g.

^n^ Total permethrins should not exceed 20 000 ng/g.

^o^ Total aflatoxin B1, B2, G1, and G2 should not exceed 20 ng/g.

^p^ Total chlordane should not exceed 100 ng/g.

Results were determined by LC‐MS/MS and LPGC‐MS/MS as indicated (n = 4).

#### 3.1.2 Method linearity, accuracy, precision, and LOQs

Based on the satisfactory performance of the selected extraction parameters which provided recoveries above 70% for all the target contaminants, the analytical figures of merit of the proposed method were evaluated. Details about the construction of calibration curves, and the assessment of method accuracy and precision are provided in the Materials and Methods section. As shown in Table 1, satisfactory results were obtained for all target analytes with good linearity (>0.99), accuracy (81‐114%), and precision (RSD values <23%). In addition, the LOQ values estimated for all the compounds comply with the requirements established by the state of California, and in the majority of the cases such LOQ values are significantly below the requested action levels [2]. Supporting information Figures S4 and S5 show LC and GC chromatograms corresponding to representative analytes at their LOQ levels, respectively. One of the most remarkable features of the herein proposed workflow is that both LC and GC instrumental methods are 12 min long. Typically, GC methods for the analysis of multiple pesticides in complex matrices require long run times to ensure that heavy chemicals are eluted from the column [23]. In our case, the use of LPGC‐MS/MS enabled a faster and reliable analysis of seven pesticides from the California list and two ISTD. It is worth emphasizing that the practical implementation of LPGC‐MS/MS is possible by employing a setup proposed by de Zeeuw et al. where the outlet of a wide analytical column is connected to the MS source, and a short and narrow restriction capillary connects the GC inlet, which is kept at positive pressure, to the analytical column [46, 47]. Based on the results of this work, cannabis labs may greatly benefit by the implementation of LPGC‐MS/MS in their routine testing of those pesticides that are more amenable to GC‐MS/MS due to poor sensitivity using ESI.

#### 3.1.3 Absolute matrix effects (ionization effects)

**FIGURE 3:**
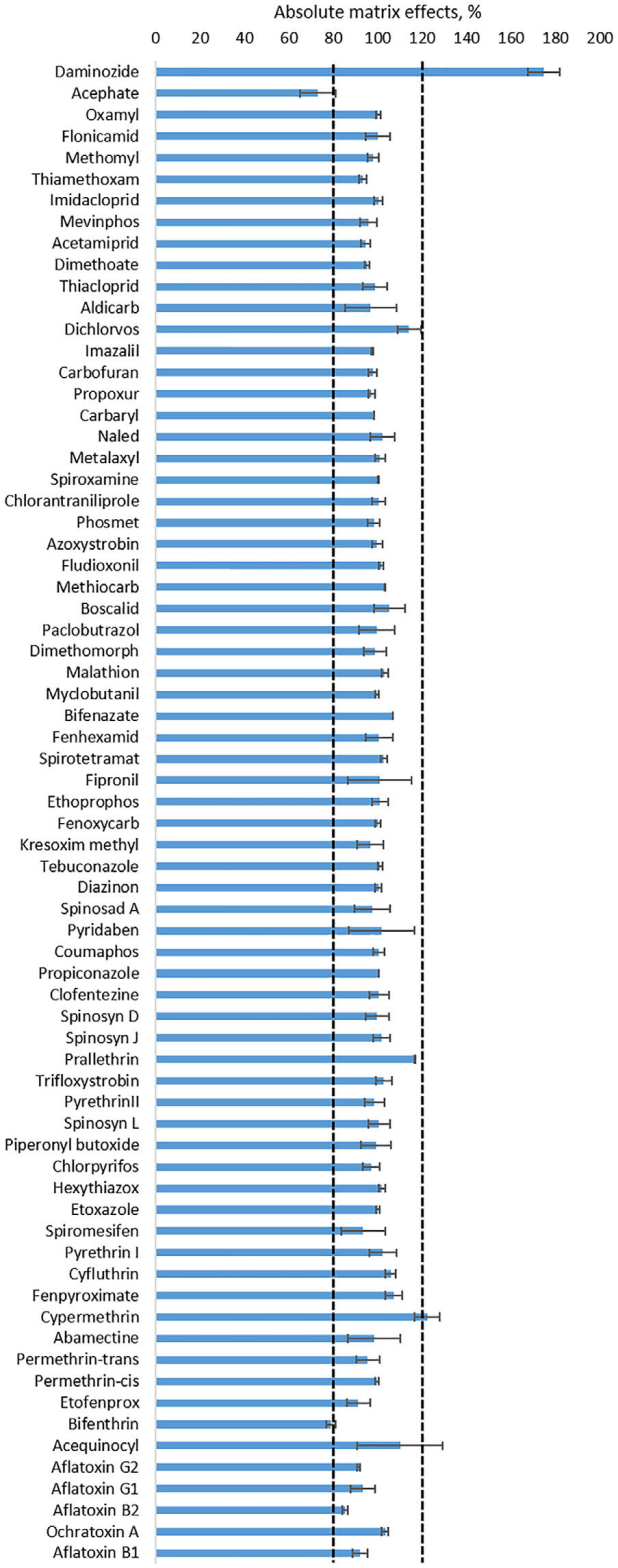
Assessment of absolute matrix effects in LC amenable pesticides (n = 3)

It is well known that ionization effects can occur in ESI when analyzing complex matrices. Coelution of matrix interferences with target analytes can lead to response suppression or enhancement depending on the nature and concentrations of both interference and analyte. Ideally, absolute matrix effect estimation should be 100%, but 80 to 120% is considered an acceptable range. Figure 3 shows the results of the evaluation of absolute matrix effects according to the procedure proposed by Matuszewski et al. [43]. As can be seen, the only compound that exhibited significant enhancement was daminozide, with 175% absolute matrix effect. Because daminozide is the most polar pesticide of the analyte list and it is not well retained under RP conditions (retention time = 0.7 min), coelution with multiple undetected polar coextractants from chocolate is likely to occur. For that reason, using appropriate matrix‐matched calibration to resemble analyte ionization conditions and/or including ISTDs that coelute with affected analytes (e.g. deuterated analogues) should be considered as strategies to account for ionization effects. In this study, the use of both matrix matched calibration and daminozide‐d6 as an ISTD enabled us to obtain reliable quantification results, with accuracy and precision values in the range of 90‐97% and 2‐4%, respectively.

#### 3.1.4 Extract stability

Results corresponding to the assessment of extract stability after 24 and 48 h of storage in the LC and GC instrument autosamplers are presented in Figure S6 (Supporting information). LC samples were stored at 10°C and GC samples were stored at room temperature. It should be noted that the final composition of the LC and GC extracts was 1:5:2 isopropanol: 1% acetic acid in acetonitrile (v/v): water and 8:92 isopropanol: 1% acetic acid in acetonitrile (v/v), respectively. Pesticides, such as flonicamid, aldicarb, dimethomorph, paclobutrazol, fipronil, propiconazole, clofentezine, pyrethrin II, piperonyl butoxide, abamectine, and chlordane, exhibited a decrease in their response within the range of 10 to 17% after 48 h of storage. Linuron‐d6 and dichlorvos‐d6, two of the ISTDs used in our workflow, also showed an approximate 10% decrease in their response. Although most of the target contaminants are stable at the proposed storage conditions and at the final extract composition chosen for this method, these findings indicate that storage time is a parameter that could significantly affect accuracy and precision of the analytical method. Setting a lower temperature in the autosampler may help to preserve the integrity of the affected analytes; however, these parameters should be further evaluated taking into account different matrices and extracts compositions.

### 3.2 Analysis of cannabinoids

Considering the importance of streamlining analytical testing of various groups of analytes in complex samples, the feasibility of using the same extract for potency testing was evaluated. Since cannabinoids are hydrophobic compounds present at high concentrations in cannabis products, the best strategy was considered to be a dilution of the organic extract for further quantitation.

Results corresponding to the first trials where chocolate samples were spiked with CBD, CBN, and delta 9‐THC at a concentration level of 0.6 mg/g showed recoveries of 105 (±12)%, 99 (±13)%, and 105 (±14)% (n = 3) for the three cannabinoids, respectively. Based on these findings, extractions from chocolate infused with CBD at a concentration of 1 mg/g were conducted using different volumes of extraction solvent. The main purpose of this experiment was to verify that 3 mL was sufficient to obtain reliable potency data. Table 2 presents the extraction recoveries estimated using 3, 6, and 12 mL of solvent. As shown, equivalent CBD concentrations were found at the three solvent volumes tested (one‐way‐ANOVA, *F* = 4.5, *F* crit = 9.55, α = 0.05). These preliminary experiments were conducted using LC‐MS/MS because that instrument was available at the moment.

**TABLE 2:** CBD recoveries obtained with 3, 6, and 12 mL of solvent from CBD‐infused chocolate (1 mg/g) (n = 2)

Total solvent volume, mL	IPA, mL	ACN + 1% AA, mL	Estimated concentration	% Difference (label comparison)
3.0	0.5	2.5	1.1 ± 0.01	6.2
6.0	1.0	5.0	0.9 ± 0.01	5.2
12	2.0	10	0.9 ± 0.04	7.3

Following these initial tests, a thorough method was developed by using HPLC‐UV as instrumental platform and including the six cannabinoids required for testing in the state of California. It is worth emphasizing that HPLC‐UV is the preferred instrumental approach for potency testing in most cannabis labs due to its robustness and affordability. Table 3 summarizes the results corresponding to method accuracy and precision determined at three concentration levels (0.2, 0.5, and 1 mg/g). As can be seen, all the cannabinoids were successfully quantified at the three spiked concentrations by employing a calibration curve prepared in solvent. This demonstrates the suitability of the proposed approach to use the same extract to quantify not only pesticides and mycotoxins, but also cannabinoids. Previous work published by Dawson et al. documented variable recoveries of cannabinoids from chocolate when varying amounts of sample were extracted with 20 mL of methanol, followed by vortexing, sonication, centrifugation, winterization, and filtration [32]. In another study, Favell et al. reported the analysis of 14 cannabinoids from various chocolate types by applying an ultrasonic extraction in acetonitrile, followed by a winterization step to remove waxes [41]. Although our work did not assess in depth the effects of multiple parameters, such as chocolate type and amount, among others, the sample preparation conditions proposed in this workflow provided promising results for the selected target cannabinoids. Future work should consider the assessment of the proposed method for the analysis of a larger list of cannabinoids according to the SMPRs recommended by the AOAC for edible chocolate [48].

**TABLE 3:** Results corresponding to cannabinoids analysis in chocolate samples at three different concentration levels, 0.2, 0.5, and 1 mg/g (n = 3)

Compounds	Retention time, min	*R* ^2^	Spiking level 1 0.2 mg/g	Spiking level 2 0.5 mg/g	Spiking level 3 1 mg/g
CBDA	2.2	0.9986	0.2 ± 0.01	0.5 ± 0.01	1.0 ± 0.04
CBG	2.4	0.9971	0.2 ± 0.01	0.5 ± 0.02	1.0 ± 0.06
CBD	2.6	0.9982	0.2 ± 0.01	0.5 ± 0.02	1.0 ± 0.01
CBN	3.8	0.9984	0.2 ± 0.01	0.5 ± 0.01	1.0 ± 0.01
Delta 9‐THC	4.8	0.9981	0.2 ± 0.01	0.5 ± 0.01	1.0 ± 0.02
THCA‐A	6.4	0.9979	0.2 ± 0.01	0.5 ± 0.02	1.0 ± 0.03

Extracts were analyzed using HPLC‐UV.

## 4 CONCLUSIONS

In this work, an easy and robust workflow for the analysis of the California list of pesticides, mycotoxins, and cannabinoids in chocolate matrix using LC and GC‐based platforms was developed and evaluated. The proposed method involved the use of 3 mL of organic solvent, followed by SPE and dSPE clean‐up steps for the analysis of contaminants and a dilution of the original extract followed by a centrifugation step for the determination of cannabinoids. As for instrumental analysis, pesticides and mycotoxins were successfully analyzed in 12 min via LC and LPGC‐MS/MS conditions. Fast GC analysis was possible by employing an LPGC setup that enabled faster elution of analytes of interest and coextracted interferences while maintaining resolution. In terms of ionization effects, only daminozide exhibited a pronounced enhancement that could be accounted for by using its deuterated analogue. Eleven pesticides showed greater than a 10% decrease in their response after 48 h of storage in the autosamplers at 10°C (LC‐MS/MS) and room temperature (GC‐MS/MS). For this reason, special attention should be paid to the time span from extract preparation to sample analysis. Cannabinoids were analyzed under HPLC‐UV conditions and satisfactory quantitation results were attained by running calibrators prepared in neat solvent. Future work should involve further evaluation of the proposed method in different chocolate types based on the SMPRs published by the AOAC [48]. Overall, excellent results for the figures of merit, such as linearity, accuracy, precision, and LOQs, were found for all the target analytes.

## CONFLICT OF INTEREST

N.R‐G and C.M. work as applications scientists at Restek Corporation (Bellefonte, PA, USA).

## Supporting information



Supporting information
