# Supplementary material for: Analysis of the California list of pesticides, mycotoxins, and cannabinoids in chocolate using liquid chromatography and low‐pressure gas chromatography‐based platforms
Source: J Sep Sci. 2021 May 24;44(13):2564–76. doi: 10.1002/jssc.202001265 (PMC8362103; doi:10.1002/jssc.202001265)
Supplement: Supplementary file 1 — Supporting information [file JSSC-44-2564-s001.docx]

**Analysis of the California list of pesticides, mycotoxins, and cannabinoids in chocolate using liquid chromatography and low-pressure gas chromatography-based platforms**

**Supplementary Information**

Nathaly Reyes-Garces^1*^ and Colton Myers^1^

^1^Restek Corporation, 110 Benner Circle, Bellefonte, PA, USA

*Corresponding author: [nathaly.reyes@restek.com](mailto:nathaly.reyes@restek.com)

**Composition of each of the mixes containing the California list of pesticides**

**California Pesticide Standard #1 (12 components (CAS number))**

Acephate (30560-19-1); Chlorpyrifos (2921-88-2); Coumaphos (56-72-4); Diazinon (333-41-5); Dichlorvos (DDVP) (62-73-7); Dimethoate (60-51-5); Ethoprophos (13194-48-4); Malathion (121-75-5); Methyl parathion (298-00-0); Mevinphos (7786-34-7); Naled (300-76-5); Phosmet (732-11-6).

**California Pesticide Standard #2 (11 components (CAS number))**

Abamectin (71751-41-2); Acequinocyl (57960-19-7); Bifenthrin (82657-04-3); Cyfluthrin (68359-37-5); Cypermethrin (52315-07-8); Etofenprox (80844-07-1); Permethrin (cis & trans) (52645-53-1); Prallethrin (23031-36-9); Pyrethrins (8003-34-7); Spinetoram (J&L) (935545-74-7); Spinosad (168316-95-8).

**California Pesticide Standard #3 (9 components (CAS number))**

Aldicarb (116-06-3); Bifenazate (149877-41-8); Carbaryl (Sevin) (63-25-2); Carbofuran (1563-66-2); Fenoxycarb (72490-01-8); Methiocarb (2032-65-7); Methomyl (16752-77-5); Oxamyl (23135-22-0); Propoxur (Baygon) (114-26-1).

**California Pesticide Standard #4 (9 components (CAS number))**

Boscalid (188425-85-6); Captan (133-06-2); Chlorantraniliprole (500008-45-7); Daminozide (1596-84-5); Dimethomorph (110488-70-5); Fenhexamid (126833-17-8); Flonicamid (158062-67-0); Hexythiazox (78587-05-0); Pyridaben (96489-71-3)

**California Pesticide Standard #5 (10 components (CAS number))**

Azoxystrobin (131860-33-8); Chlorfenapyr (122453-73-0); Fenpyroximate (111812-58-9); Kresoxim methyl (143390-89-0); Metalaxyl (57837-19-1); Piperonyl butoxide (51-03-6); Spiromesifen (283594-90-1); Spirotetramat (203313-25-1); Spiroxamine (118134-30-8); Trifloxystrobin (141517-21-7).

**California Pesticide Standard #6 (15 components (CAS number))**

Acetamiprid (135410-20-7); Chlordane (57-74-9); Clofentezine (74115-24-5); Etoxazole (153233-91-1); Fipronil (120068-37-3); Fludioxonil (131341-86-1); Imazalil (35554-44-0); Imidacloprid (138261-41-3); Myclobutanil (88671-89-0); Paclobutrazol (76738-62-0); Pentachloronitrobenzene (Quintozene) (82-68-8); Propiconazole (Tilt) (60207-90-1); Tebuconazole (107534-96-3); Thiacloprid (111988-49-9); Thiamethoxam (153719-23-4).

**Table S1.** LC-MS/MS transitions monitored for the analysis of pesticides and mycotoxins.

| **Name** | **Retention time, min** | **Precursor ion** | **Product ion 1** | **Product ion 2** |
| --- | --- | --- | --- | --- |
| Daminozide-D6 | 0.7 | 167.0 | 149.3 | 49.3 |
| Daminozide | 0.7 | 161.1 | 44.1 | 143.2 |
| Acephate | 1.7 | 184.0 | 143.1 | 95.1 |
| Oxamyl | 2.0 | 237.1 | 72.1 | 90.1 |
| Flonicamid | 2.1 | 230.1 | 203.1 | 174.1 |
| Methomyl | 2.1 | 163.1 | 88.1 | 106.1 |
| Thiamethoxam | 2.1 | 292.0 | 211.1 | 181.1 |
| Imidacloprid | 2.3 | 256.1 | 209.1 | 175.1 |
| Mevinphos | 2.4 | 225.1 | 127.1 | 193.2 |
| Acetamiprid | 2.4 | 223.0 | 126.1 | 56.1 |
| Dimethoathe-D6 | 2.4 | 236.1 | 205.1 |  |
| Dimethoate | 2.4 | 230.0 | 199.1 | 125.1 |
| Thiacloprid | 2.5 | 253.0 | 126.0 | 90.1 |
| Aflatoxin G2 | 2.5 | 331.2 | 189.3 | 115.2 |
| Aflatoxin G1 | 2.5 | 329.2 | 243.2 | 215.3 |
| Aldicarb | 2.6 | 116.0 | 89.2 | 70.2 |
| Aflatoxin B2 | 2.6 | 315.3 | 287.2 | 243.3 |
| Dichlorvos | 2.7 | 220.9 | 109.1 | 79.2 |
| Dichlorvos-D6 | 2.7 | 227.0 | 115.1 |  |
| Aflatoxin B1 | 2.7 | 313.2 | 241.2 | 128.2 |
| Imazalil | 2.7 | 297.0 | 159.0 | 201.0 |
| Carbofuran | 2.7 | 222.1 | 123.1 | 165.2 |
| Propoxur | 2.7 | 210.1 | 111.1 | 93.1 |
| Carbaryl-D7 | 2.8 | 209.2 | 152.2 |  |
| Carbaryl | 2.8 | 202.1 | 145.1 | 127.1 |
| Diuron-D6 | 3.0 | 239.1 | 78.2 |  |
| Atrazine-D5 | 3.0 | 221.2 | 179.1 |  |
| Naled | 3.1 | 397.8 | 127.1 | 109.1 |
| Metalaxyl | 3.1 | 280.2 | 220.2 | 192.2 |
| Spiroxamine | 3.1 | 298.3 | 144.2 | 100.2 |
| Chlorantraniliprole | 3.2 | 483.9 | 452.9 | 285.9 |
| Phosmet | 3.2 | 318.0 | 160.1 | 77.2 |
| Azoxystrobin | 3.3 | 404.0 | 372.1 | 344.1 |
| Linuron-D6 | 3.3 | 255.1 | 160.1 |  |
| Fludioxonil* | 3.4 | 247.0 | 180.0 | 126.0 |
| Methiocarb | 3.4 | 226.1 | 169.1 | 121.1 |
| Dimethomorph | 3.5 | 388.2 | 301.2 | 165.3 |
| Boscalid | 3.5 | 342.9 | 307.1 | 140.1 |
| Paclobutrazol | 3.6 | 294.3 | 70.1 | 125.1 |
| Malathion | 3.6 | 331.0 | 127.2 | 285.2 |
| Myclobutanil | 3.7 | 289.1 | 70.1 | 125.1 |
| Bifenazate | 3.7 | 301.0 | 198.1 | 170.2 |
| Ochratoxin A | 3.8 | 404.2 | 239.1 | 358.3 |
| Fenhexamid | 3.9 | 302.1 | 97.1 | 55.2 |
| Spirotetramat | 4.0 | 374.2 | 302.1 | 216.1 |
| Ethoprophos | 4.1 | 243.1 | 131.1 | 97.1 |
| Fipronil* | 4.1 | 436.8 | 331.8 | 251.9 |
| Fenoxycarb | 4.2 | 302.1 | 88.1 | 116.1 |
| Kresoxim methyl | 4.4 | 314.2 | 267.2 | 222.2 |
| Tebuconazole | 4.4 | 308.1 | 70.1 | 125.1 |
| Diazinon-D10 | 4.6 | 315.2 | 170.2 |  |
| Spinosad (spinosyn A) | 4.6 | 732.4 | 142.2 | 98.1 |
| Diazinon | 4.6 | 305.1 | 169.2 | 153.2 |
| Coumaphos | 4.7 | 363.1 | 227.1 | 307.1 |
| Pyridaben | 4.7 | 365.1 | 309.2 | 147.2 |
| Propiconazole | 4.7 | 342.0 | 159.0 | 69.2 |
| Clofentezine | 4.8 | 303.0 | 138.1 | 102.1 |
| Spinosad (spinosyn D) | 5.0 | 746.5 | 142.3 | 98.4 |
| Spinetoram (spinosyn J) | 5.1 | 748.5 | 142.3 | 98.3 |
| Trifloxystrobin | 5.3 | 409.2 | 186.1 | 145.1 |
| Prallethrin | 5.3 | 301.2 | 123.2 | 105.2 |
| Pyrethrin II | 5.5 | 373.1 | 161.1 | 133.2 |
| Spinetoram (spinosyn L) | 5.6 | 760.5 | 142.2 | 98.1 |
| Piperonyl butoxide | 6.0 | 356.3 | 177.2 | 119.2 |
| Chlorpyrifos | 6.1 | 349.9 | 198.0 | 97.1 |
| Hexythiazox | 6.2 | 353.1 | 228.1 | 168.1 |
| Etoxazole | 6.6 | 360.2 | 141.1 | 304.2 |
| Spiromesifen | 6.7 | 273.2 | 255.2 | 187.2 |
| Pyrethrin I | 6.9 | 329.2 | 161.2 | 105.2 |
| Cyfluthrin (qualifier) | 6.9 | 453.1 | 193.2 |  |
| Cyfluthrin | 6.9 | 451.1 | 191.2 |  |
| Cypermethrin | 7.1 | 433.1 | 191.0 | 416.0 |
| Fenpyroximate | 7.1 | 422.2 | 366.1 | 138.1 |
| Permethrin-trans | 7.6 | 408.3 | 183.2 | 355.1 |
| Permethrin-cis | 7.9 | 408.3 | 183.2 | 355.1 |
| Abamectine | 7.9 | 890.5 | 305.4 | 567.4 |
| Etofenprox | 8.0 | 394.3 | 177.2 | 359.3 |
| Bifenthrin | 8.2 | 440.0 | 181.2 | 166.2 |
| Acequinocyl 1 | 9.4 | 402.3 | 343.2 | 189.0 |
| Acequinocyl 2 | 9.4 | 386.0 | 344.2 | 189.1 |

**Figure S1.** Representative chromatogram corresponding to the LC-MS/MS analysis of pesticides and mycotoxins in a chocolate sample spiked at 100 ng/g.

**Table S2.** LC-MS/MS transitions monitored for the analysis of cannabinoids

| **Name** | **Retention time, min** | **Precursor ion** | **Product ion 1** | **Product ion 2** |
| --- | --- | --- | --- | --- |
| Cannabidiol (CBD) | 2.5 | 315.3 | 193.0 | 123.1 |
| Cannabinol (CBN) | 3.8 | 311.3 | 223.3 | 293.3 |
| delta 9-tetrahydrocannabinol (delta 9-THC) | 4.8 | 315.3 | 193.0 | 123.1 |

**Table S3.** LPGC-MS/MS parameters

| Thermo Trace 1310 / TSQ 8000 Parameters | |
| --- | --- |
| LPGC Column | Rtx-5ms 15m x 0.53mm x 1.0µm w/ Hydroguard 5m x 0.18mm |
| Injection | Splitless |
| Inj. Vol. | 1 µL |
| Liner | Topaz 4.0 mm Single Taper Inlet Liner w/ Wool (cat# 23447) |
| Inj. Temp. | 250 °C |
| Split Flow | 20 mL/min |
| Purge Flow | 5.0 mL/min |
| Splitless Time | 0.50 min |
| Oven | 80 °C (hold 1 min) to 330 °C (hold 5.50 min) by 45 °C/min |
| Carrier Gas | He, constant flow |
| Flow Rate | 2.0 mL/min |
| Detector | MS/MS |
| Mode | Acquisition - Timed |
| Transfer Line Temp. | 290 °C |
| Ion Source Temp. | 325 °C |
| Ionization Mode | EI |
| Total Scan Time | 0.300 sec |
| SRM/SIM Time | 0.300 sec |
| Min. Baseline Peak Width | 3.0 sec |
| Desired Scans Per Peak | 10 |

**Table S4.** GC-MS/MS transitions monitored for the analysis of selected pesticides

| **Name** | **Retention time, min** | **Ion Polarity** | **Precursor ion** | **Product ion** |
| --- | --- | --- | --- | --- |
| Atrazine-D5 (IS) (Quan) | 4.57 | Positive | 220.0 | 58.0 |
| Atrazine-D5 (IS) (Qual) | 4.57 | Positive | 205.0 | 127.0 |
| Diazinon-D10 (Quan) | 4.65 | Positive | 183.0 | 139.0 |
| Diazinon-D10 (Qual) | 4.65 | Positive | 183.0 | 168.0 |
| Quintozene (PCNB) (Quan) | 4.70 | Positive | 294.9 | 236.9 |
| Quintozene (PCNB) (Qual) | 4.70 | Positive | 236.8 | 118.9 |
| Methyl Parathion (Quan) | 4.96 | Positive | 263.0 | 109.0 |
| Methyl Parathion (Qual) | 4.96 | Positive | 263.0 | 79.0 |
| Captan (Quan) | 5.47 | Positive | 184.0 | 149.1 |
| Captan (Qual) | 5.47 | Positive | 184.0 | 134.1 |
| Chlordane (cis and trans) (Quan) | 5.56 | Positive | 271.9 | 237.0 |
| Chlordane (cis and trans) (Qual) | 5.56 | Positive | 372.9 | 265.9 |
| Chlorfenapyr (Quan) | 5.69 | Positive | 247.1 | 227.1 |
| Chlorfenapyr (Qual) | 5.69 | Positive | 59.1 | 31.1 |
| Cyfluthrin (Quan) | 6.71 | Positive | 226.0 | 206.0 |
| Cyfluthrin (Qual) | 6.71 | Positive | 163.0 | 127.0 |
| Cypermethrin (Quan) | 6.80 | Positive | 163.0 | 127.1 |
| Cypermethrin (Qual) | 6.80 | Positive | 181.1 | 152.1 |


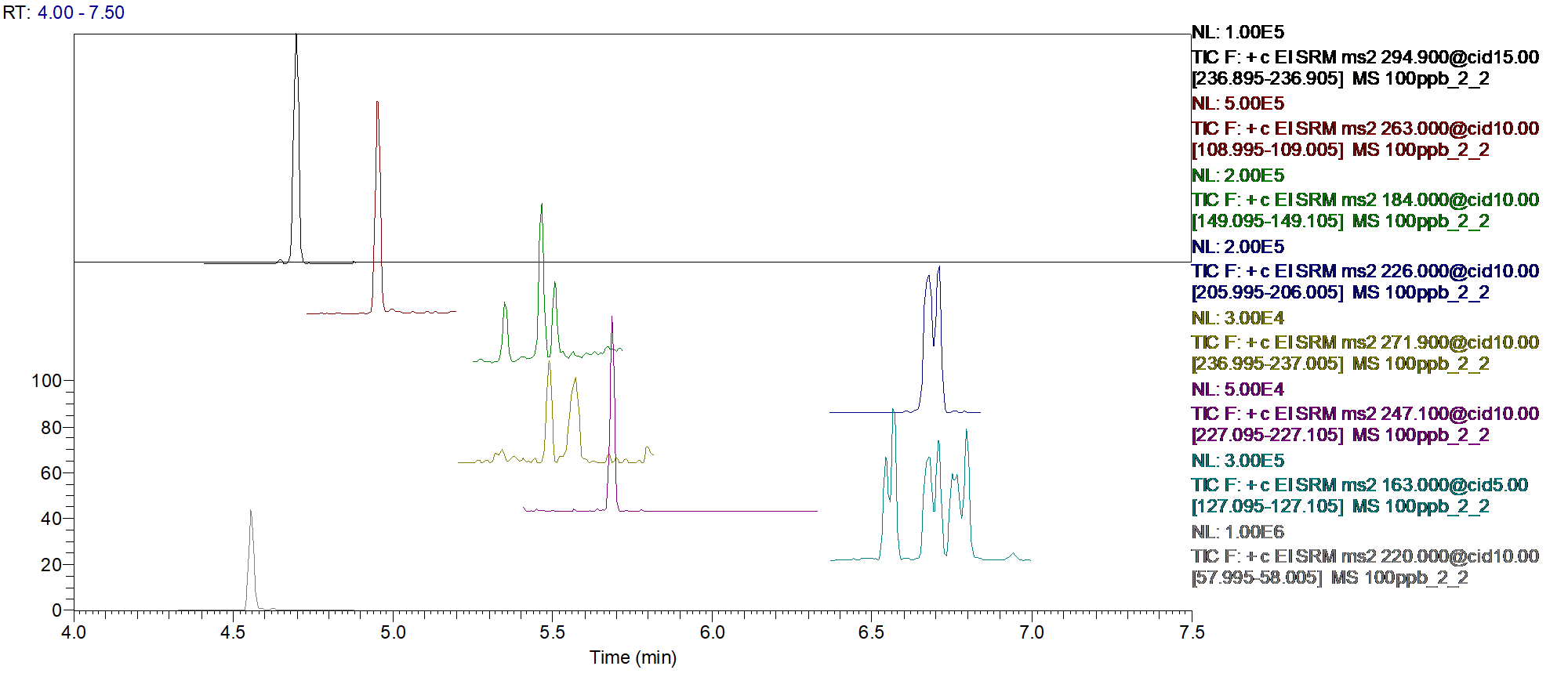


**Figure S2.** Representative chromatogram corresponding to the LPGC-MS/MS analysis of pesticides in a chocolate sample spiked at 100 ng/g.

**Figure S3.** Chromatogram corresponding to the analysis of cannabinoids in a chocolate sample via HPLC-UV (228 nm). Analytes were spiked at 0.2 mg/g. 1. CBDA (2.2 min); 2. CBG (2.4 min); 3. CBD (2.6 min); 4. CBN (3.8 min); 5. Delta 9-THC (4.8); 6. THCA-A (6.4 min).

**Figure S4.** Chromatograms corresponding to GC-amenable pesticides at their LOQ levels. A) PCNB; B) methylparathion; C) captan; D) trans-chlordane; E) cis-chlordane; F) chlorfenapyr; G) cyfluthrin and H) cypermethrin.

**Figure S5.** Chromatograms corresponding to representative LC-amenable contaminants at their LOQ levels. A) daminozide; B) oxamyl; C) imazalil; D) spiroxamine; E) methiocarb; F) fipronil; G) fenoxycarb; H) chlorpyrifos; I) aflatoxin G1; J) aflatoxin B2; K) aflatoxin B1 and L) ochratoxin A.

**Figure S6.** Assessment of stability of pesticides and mycotoxins samples after 24 and 48 hours of storage in the autosamplers (analytes tested via GC-MS/MS are marked with *) (n=3). Samples for LC-MS/MS analysis were stored at 10 ⁰C, and samples for GC-MS/MS analysis were stored at room temperature. Responses normalized by areas obtained from fresh extracts (0 h).
